# The opioid epidemic: a central role for the blood brain barrier in opioid analgesia and abuse

**DOI:** 10.1186/s12987-017-0080-3

**Published:** 2017-11-29

**Authors:** Charles P. Schaefer, Margaret E. Tome, Thomas P. Davis

**Affiliations:** Department of Pharmacology, University of Arizona, P.O. Box 245050, Tucson, AZ 85724 USA

**Keywords:** Opioids, Morphine, Pain, Blood–brain barrier, P-Glycoprotein, Opioid tolerance

## Abstract

Opioids are currently the primary treatment method used to manage both acute and chronic pain. In the past two to three decades, there has been a surge in the use, abuse and misuse of opioids. The mechanism by which opioids relieve pain and induce euphoria is dependent on the drug crossing the blood–brain barrier and accessing the central nervous system. This suggests the blood brain barrier plays a central role in both the benefits and risks of opioid use. The complex physiological responses to opioids that provide the benefits and drive the abuse also needs to be considered in the resolution of the opioid epidemic.

## Background

In the United States, the abuse of opioids is currently described as an epidemic. On average, 3900 individuals begin the non-medical use of prescription opioids, and 580 individuals begin heroin use every day [1]. Drug overdose deaths related to opioids, including both opioid pain relievers and heroin, increased 200% between 2000 and 2014 [2]. This trend is continuing unabated. Yet, opioids are the most effective therapy for reducing reported pain in most patients. For example, pain management is an important component of post-surgical recovery. Poor pain management can impair recovery, increase the probability of readmission, increase the cost of care and decrease patient satisfaction [3]. Intravenous opioid analgesics, such as morphine, are currently the standard of care for post-surgical pain. The yin and yang of the opioid response leads to the clinical challenge of how to treat short/moderate duration post-surgical pain without causing opioid dependence that could lead to abuse.

The purpose of this review is to first, trace the history of the use and abuse of opioids and put this into the context of our current understanding of the physiology of pain. Next, we examine the role that the blood brain barrier (BBB) plays in opioid analgesia and euphoria. We have highlighted the central role of the BBB in opioid analgesia and abuse because it is a critical regulator of opioid access to the central nervous system (CNS).

### Physiology of pain

In his famous novel *1984*, George Orwell describes “Of pain you could wish only one thing: that it should stop. Nothing in the world was so bad as physical pain. In the face of pain there are no heroes”.

Pain and the negative emotions associated with it serve as invaluable tools for survival. Acute pain acts as a signal of noxious stimuli and the negative emotional response associated with the pain reinforces behaviors that avoid these stimuli. Persistent pain acts as a clue of internal injuries such as muscular damage or broken bones. Changes can occur in pain pathways resulting in an altered, chronic state. When chronic pain is associated with an injury, this can alter behavior to protect the site of an injury allowing the injury to heal without further harm. In some cases, chronic pain will persist at the site of an injury well past the time protective pain is beneficial to healing.

The physical component of pain, nociception, is the process by which nociceptors, a group of nerve cells found in the peripheral nervous system, recognize intense thermal, mechanical or chemical stimuli [4]. Nociceptors have a unique physiology; they have cell bodies in specific regions known as ganglia. In the periphery, the cell bodies of nociceptors are located in the dorsal root ganglion. Nociceptors have two axonal branches, a peripheral branch that innervates the target organ and a central axon that innervates the spinal cord [5]. A key feature of nociceptors is the ability to limit the initiation of a signal in response to noxious stimuli by requiring a relatively high activation signal. Nociceptors are divided into two groups of fibers. The Aδ-fibers and Aβ-fibers are thinly myelinated fibers responsible for transmitting “acute, well-localized, fast pain,” specifying the location of the stimulus [4]. The second type of fiber is the unmyelinated C-fiber which is responsible for poorly-localized “slow” pain often described as an ache. Both of these fiber types can be organized into subtypes that are more or less sensitive to thermal or mechanical stimulation.

In the central nervous system, nociceptors project to differing laminae of the dorsal horn of the spinal cord depending on the type of nociceptive fiber. A variety of signaling molecules act at the synapses between the central terminal of the nociceptors and the laminae of the spinal cord [5]. Neurons within these laminae are responsible for transmitting the nociceptive signal through the spinal cord in a contralateral manner to the thalamus of the brain. From here, signals are sent to the somatosensory cortex and limbic system. While this process is short-lived for acute pain, persistent or chronic pain can arise when there is an anomaly in this system. The anomaly can be caused by either over sensitization or spontaneous firing of nociceptors. Pharmacological modification of this pathway is used as a strategy to reduce or eliminate pain.

### History of opioid use for pain treatment

Opioids are a key drug in our arsenal for the treatment of pain. However, the addictive and destructive properties of opioids and their derivatives present both a clinical challenge and a public health problem that we have yet to resolve. The exact origins of the use of opium for pain treatment are not known. The original use of opium was probably as a euphoriant in religious ceremonies as described in pictographs from ancient Sumerian sites. Knowledge of the process used to isolate opium was likely limited to priests [6]. Brownstein states that the earliest written records of medicinal use of the opium poppy date back to the dawn of human civilization [6]. The Sumerians were the first people to record the production and use of opium. Clay tablets dating around 3000 BC describe the process by which the opium poppy was cultivated. The tablets also describe how to extract the juice from the cultivated flowers and the process by which this juice is processed into opium. Cultivation of this plant remained popular, spanning many centuries and empires and eventually led to the distribution of opium throughout Eurasia.

The complex issues surrounding opioid use are illustrated by the history of opium use in China. As documented in Schiff, Arabian traders brought opium and knowledge of the medicinal use of the drug to the country at some point between the 11th and 13th centuries AD [7]. This review goes on to say that following a ban on smoking tobacco by Tsung Chen in 1644, smoking opium became a popular replacement for many Chinese citizens. Opium sold in China originated from large growing operations in India distributed by the East India Company. Following the acquisition of the East India Company by the British government, large quantities of opium were sold to smaller companies that would smuggle the drug into China. These companies sold the opium through Canton. Following the replacement of the Viceroy of Canton in 1838, opium distribution was severely reduced. In 1839, millions of pounds of British and American opium were confiscated and destroyed by the Viceroy. This sparked the first opium war resulting in Britain being awarded control of the island of Hong Kong for over 150 years. By 1913, 25% of the Chinese population was addicted to opium. This epidemic prompted the British government to suspend the sale of opium, but this action came too late. Widespread use of opium would not stop in China until the years following World War II with the establishment of the People’s Republic of China.

The search for opioid derivatives that retain efficacy and decrease addiction also has a long history. In 1806, morphine was isolated from the opium poppy by Sertüner [8]. Morphine could be produced in large quantities and became popular to use for minor surgical procedures and for the management of post-surgical and chronic pain. This discovery was not the solution for opiate addiction that many had hoped for and triggered the widespread search for a non-addictive replacement. In 1898, heroin was first synthesized with the claim of being more potent than morphine and being free from an addictive nature like other opioids [9]. Only one of these claims would prove to be true, and both heroin abuse and the search for a non-addictive opioid continue today [6]. The search for a non-addictive replacement resulted in the synthesis of methadone in 1946 which led to the first potential treatment for opioid addiction [10]. The symptoms of withdrawal syndrome associated with methadone use were markedly more manageable than those associated with traditional opioids. While these symptoms have a longer duration, the effects experienced are milder. This observation inspired a treatment plan in which patients would be switched from an opioid to methadone with the goal that administration would be tapered off entirely [6]. These programs rely on very careful monitoring of drug intake combined with the addition of supportive behavioral therapies and lead to lowered mortality rates than in those who do not use this therapy [11]. Those using this therapy are also able to maintain mostly normal lives, easing the transition out of addiction [6].

Use of opioids for pain management has waxed and waned through history in part because of changing attitudes toward the risk/benefit balance of such treatment. For example, chronic opioid therapy for non-cancer related chronic pain has been a standard use of these drugs throughout history. While this did fall out of favor though much of the 20th century due to the danger of addiction and other adverse effects, attitudes began to change in the 1980s [12]. A letter written to the New England Journal of Medicine made a significant impact on attitudes towards the addictive nature of opioids in chronic pain patients [13]. The letter explained that of the 11,882 examined patients who received at least one prescription of a narcotic, only four had well-documented addiction after leaving the hospital. The feeling of safety related to chronic opioid use was further reinforced by letters and scholarly reviews throughout the following decades. These studies often involved patients with a history of opioid use presenting little to no evidence of addiction [14–16]. Of these studies, an article published in *Pain* was particularly notable. This study followed 38 patients who had received opioids for an extended period reporting misuse in only two patients [15]. This gave the impression that if an opioid was prescribed for pain, there was little danger of addiction. The shift in attitudes towards opioids as a complete solution for all types of pain management seemed to answer the increasing demand for pain management in clinical settings [12]. The relaxed attitudes surrounding opioids began to be questioned again after a decade long trend, beginning in 2000, resulted in large changes of opioid use. Articles and reviews were published detailing the increase in opioid prescriptions across all types of clinical settings [17, 18]. Increasing trends in opioid use, as well as the increase in opioid prescriptions, are currently raising public safety concerns.

### Physiology of the opioid response: crossing the blood brain barrier

Opioids are a class of drugs with several useful effects including cough suppression, gastric slowing, and as they are most commonly prescribed, analgesia. Opioid analgesics can be administered through suppository or intrathecally, intravenously, or orally. More lipophilic opioids can also be administered transdermally. As described by Yaksh and Wallace in *Goodman and Gilman’s: The Pharmacological Basis of Therapeutics*, oral opioids are subject to the first pass effect in the liver as well as poor absorption due to gastric ion trapping and have a bioavailability of about 25% [19]. Intravenous administration of opioids results in prompt action [19]. The speed of action is affected by the lipophilicity of the compound which contributes to differences in the speed at which the compound can cross the BBB and enter the CNS. Morphine does not persist in tissue and is found in trace quantities 24 h after the last administered dose. Metabolism of morphine relies on conjugation with glucuronic acid producing two metabolites, morphine-6-glucuronide (M6G) and morphine-3-glucuronide (M3G). M6G has an analgesic effect. It is twice as potent as morphine, and is thought to make up a significant portion of morphine’s analgesic effect in patients treated with long-term opioid therapy [20]. The more prevalent metabolite, M3G, is known to have neuroexcitatory effects [21]. M3G is also the primary form excreted from the body [19]. While almost no unmodified morphine is excreted, morphine’s metabolites are excreted through the kidneys.

The analgesic effect of opioids is due to pharmacological action in the brain, in the spinal cord, and potentially in the periphery. In the brain, opioids act at mu opioid receptors (MOR). Mutations in the MOR at position 118 are sufficient to modify post-cesarean pain perceptions and the amount of morphine used by patients through a patient-controlled analgesia system [22]. Experiments involving microinjections at the medulla, substantia nigra, nucleus accumbens, and periaqueductal gray (PAG) resulted in the reduction of pain behaviors in animal models [23]. The action in the PAG causes a disinhibition of the medulla at tonically active neurons [19]. This disinhibition leads to the release of norepinephrine and serotonin to the spinal dorsal horn, attenuating dorsal horn excitability [23]. This attenuation results in a reduction of nociceptive signaling through the spinal cord.

### The blood–brain barrier: opioid access to the CNS and the role of P-glycoprotein

The main analgesic response to opioids occurs at the level of the CNS. To exert this effect, the opioids must cross the BBB. The BBB serves as a selectively permeable physical and biochemical barrier that contributes to the maintenance of the ionic homeostatic environment required for proper neuronal function in the CNS. Evolutionary studies have shown that this type of barrier was essential for the development and function of increasing complex brains in vertebrates [24, 25]. The BBB also plays a major role in protecting the CNS from pathogens and toxins in the bloodstream. The ability of the BBB to exclude xenobiotics from the CNS serves as a challenge for delivery of pharmacological agents, including opioids, to the brain [26, 27].

**Fig. 1:**
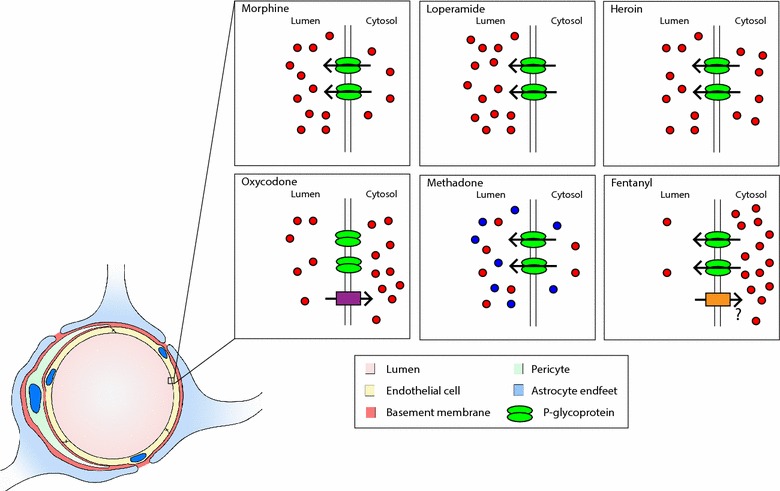
Model of the of the neurovascular unit with a diagram indicating the relative brain penetrance of selected opioids. Endothelial cells surround the capillary lumen. P-Glycoprotein (green ovals) in the luminal membrane effluxes many types of opioids (red/blue circles) back into the circulation. Additional transporters (purple/orange) at the luminal membrane transport specific opioid analogs. In the methadone figure, the red and blue circles represent different methadone enantiomers

Anatomically, the BBB is a barrier formed by endothelial cells surrounding the lumen of the brain microvasculature (Fig. 1). Adjacent endothelial cells attach themselves to each other via specific proteins forming tight junctions of high transendothelial electrical resistance. These tight junctions are made up of a complex of transmembrane proteins and prevent paracellular movement of substances from the blood into the brain [28]. Adherens junctions, which help establish cell polarity, also link endothelial cells to each other and contribute to barrier integrity. Pericytes surround the endothelial cells. Pericytes belong to the vascular smooth muscle cell family. They play an important role in the establishment of the BBB and provide structural support and maintenance signals for the mature BBB [29]. Astrocytes, which surround the endothelial cells and pericytes also contribute to BBB maintenance and regulation of barrier properties [30]. The interaction of these cell types, known as the neurovascular unit, is a critical regulator of barrier properties in response to physiological changes and under pathological conditions.

The ability of the BBB to act as a selectively permeable barrier is heavily reliant on transport proteins in the endothelial cells that regulate transcellular movement of substances. Transport proteins are essential for the movement of nutrients into the brain while keeping pathogens and toxins out. Some of the transporters are highly specific. For example, glucose, essential for brain function, requires a transporter to cross the BBB. The GLUT1 transporter is responsible for glucose transport and allows glucose to travel into the brain along its concentration gradient [31]. Some transporters act to export compounds from the BBB, most notably the ATP-Binding Cassette (ABC) proteins [32]. Of these, P-glycoprotein (P-gp), also known as multiple drug resistant protein 1 (Mdr1), plays a major role in the mechanism by which toxins and xenobiotics are excluded [33, 34]. P-gp is of particular interest because it has a wide range of substrates, including opioids, and a poorly understood system of regulators. Numerous other transporters are expressed in the BBB endothelial cells and contribute to the selective barrier properties of the BBB [26].

### The blood brain barrier: delivery of opioids to the CNS

Analgesic efficacy of opioids depends on the relative ability to cross the BBB. Opioids currently in clinical use alleviate pain mostly by binding to MOR in the CNS; uptake into the brain, therefore, is critical for efficacy. P-gp is the major drug exporter at the BBB; it is very efficient at exporting opioids [35]. In the luminal membrane, P-gp binds to drug both as it is diffusing through the endothelial cell membrane and from inside the endothelial cells [36]. It effluxes drug back into the circulation via an ATP-dependent mechanism [36]. Inhibition of P-gp to improve CNS drug delivery has not proven clinically viable because of the risk of death due to infection and toxicity [37, 38]. Therefore, an analysis of the efficacy of opioids and their derivatives depends in part on the ability of P-gp to exclude them from the CNS. An appreciation for the central role of P-gp in opioid analgesia is illustrated by the relative effects of several structurally divergent opioids (Fig. 1).

Morphine is the international standard for opioid analgesic therapy. As previously discussed, morphine is metabolized into M3G and M6G via glucuronidation, leading to blood concentrations of these metabolites several times higher than that of the parent compound. Morphine can also be metabolized to M3G and M6G in the brain directly [39]. Morphine is a substrate for P-gp [39, 40]. The analgesic efficacy of morphine is roughly proportional to the concentration of morphine in the blood and the amount of active P-gp at the BBB [41]. M6G, the metabolite with higher analgesic potency than the parent compound, is not a P-gp substrate, but may be a substrate of other transporters at the BBB [42, 43]. Genetic polymorphisms in ABCB1, the gene which encodes P-gp, in cancer patients play a major role in intracellular concentrations of morphine and both metabolites [42]. Inhibition of P-gp at the time of administration of morphine increases the observed analgesic effect, confirming P-gp inhibits the analgesic effect of morphine [44]. Multidrug resistance protein 3 effects the transport of morphine metabolites; additional studies to determine whether morphine metabolites are substrates of other members of the MDR protein family are needed [45].

Loperamide is a synthetic MOR agonist that is a stronger P-gp substrate than morphine, leading to its clinical use as an anti-diarrheal [46]. Both in vitro and in vivo models indicate that P-gp efficiently effluxes loperamide [46, 47]. The brain penetrance is minimal in humans; loperamide is marketed as an anti-diarrheal because the major effect is opioid-mediated constipation in the GI track [48]. P-gp knockout mice accumulated loperamide in the CNS and displayed opioid-mediated effects [49]. These data indicate that P-gp, by regulating brain uptake of loperamide, determines the analgesic efficacy of loperamide.

Heroin has a potency twofold greater than morphine and crosses the BBB more readily than morphine [50]. Although heroin is similar in structure to morphine, this drug is acetylated and therefore more lipophilic than morphine leading to an increased potency. Heroin is metabolized into 6-monoacetylmorphine (6-MAM) and subsequently to morphine in the blood [51]. In a study by Seleman et al. in which the effect of a P-gp inhibitor co-administered with heroin, 6-MAM, and morphine, only morphine transport was increased [27]. This study showed the transport of heroin and 6-MAM were unaffected by P-gp inhibition, suggesting that this may also play a role in the higher potency of heroin over morphine. 6-MAM has been shown to have an even greater affinity for MOR than morphine and a greater analgesic effect [51]. 6-MAM has a short half-life in humans and is rapidly metabolized into morphine. Although heroin and 6-MAM can enter the BBB, P-gp still plays a role in the effect of heroin on the CNS because of the rapid metabolism to morphine [52].

Oxycodone is a potent opioid often prescribed to manage moderate to severe pain. When co-administered with the P-gp inhibitor valspodar, transport of oxycodone into the brain was not affected [53]. Oxycodone has a lower affinity for MOR than morphine, but in similar doses is as effective as morphine in the management of post-surgical pain [54]. This prompted experimentation examining the relative BBB transport of oxycodone into the brain compared with morphine. Oxycodone is transported into the mouse brain in concentrations six times higher than morphine [35]. The relationship between the BBB and oxycodone is unique because oxycodone can be found at concentrations three times higher in the brain than in the blood [55]. A cation/H+ antiporter in the BBB endothelial cells has been implicated in the uptake of oxycodone into the brain [27, 56].

Methadone is a synthetic opioid that is used in the treatment of, especially, chronic pain and for opioid dependence [11, 57]. Methadone has lesser side effects than many other opioids, so chronic administration is often considered more manageable than for other opioids [11]. Methadone is administered as a racemic mixture of both the *R*- and *S*-enantiomers of the drug [58]. Methadone is metabolized into the pharmacologically inactive compound 2-ethylidene-1,5-dimethyl-3,3-diphenylpyrrolidine (EDDP) [59]. A study by Wang et al. showed both the *R*- and *S*-enantiomers of methadone are substrates of P-gp, limiting the delivery of the clinically used racemic methadone across the BBB [58]. This study compared concentrations of methadone found in multiple tissues throughout the body in wild-type and ABCB1a (the gene encoding P-gp in mice) knockout animals. Significantly higher concentrations of both enantiomers of methadone occurred only in the brain. Although there is minimal stereoselectivity of P-gp for methadone enantiomers, resulting in similar brain penetrance of both enantiomers, the (*R*)-enantiomer (levomethadone) is responsible for the action of methadone as a MOR agonist [40, 58, 60–62].

Fentanyl is a synthetic opioid with a potency 100-fold greater than morphine [63]. Fentanyl has become important due to its contribution to the epidemic of opioid related deaths [1]. A study by Henthorn et al. in which the CNS uptake of radiolabeled fentanyl was quantified, demonstrated that the presence of a P-gp inhibitor increased transport across bovine brain endothelial cells (an in vitro model of the BBB) [64]. This study also demonstrated that there is likely a transporter that contributes to direct transport of fentanyl across these endothelial cells. A study by Wandel et al. demonstrated that cells with an increased expression of P-gp did not have significantly lower transport of fentanyl across endothelial cells in vitro [46]. This suggests that other components of the neurovascular unit may play a significant role in fentanyl transport at the BBB. Further investigation into this mechanism would provide a path to reducing the dangers and addictive nature of this drug.

Analgesic efficacy of the opioids is complicated by additional factors in the clinic. The comparison studies on the relative ability of P-gp to efflux opioids are based on the same genetic variant of P-gp. Genetic polymorphisms in P-gp will affect the amount of drug excluded from the CNS at a given dose in humans [65]. As mentioned above, the ability of opioids (or their active metabolites) to cross the BBB can also depend on other transport mechanisms, as suggested by the data on oxycodone [56]. Once in the brain, the relative binding to MOR, rate of metabolism of the native compound, and relative activity of the metabolites will all contribute to analgesic efficacy (e.g., [52, 54]). Genetic polymorphisms that alter proteins in these pathways increase the difficulty of predicting the analgesic efficacy for a given patient [65]. In this review, we have chosen to discuss a few opioids in detail to illustrate the central role of the BBB. Many additional opioid derivatives exist. The complexity illustrated by our examples, however, indicates the extent to which opioids need to be studied to determine their best clinical use; an analysis of their ability to cross the BBB is an important component.

### The blood brain barrier: opioid-induced euphoria

Opioid transport across the BBB into the CNS is essential for the euphoric effects of opioids [19]. A review by Xi and Stein summarizes the reward associated with opioids as, disinhibition of GABAergic neurons in the nucleus accumbens by dopaminergic neurons from the ventral tegmental area (VTA) which increases activity in the ventral pallidum and causes an increase of dopamine release [66]. Animals with the ability to deliver morphine directly to the VTA will continue to do so [66]. This suggests a feeling of reward for the animal and the presence of opioids in the brain is therefore capable of eliciting this response. This response to the presence of morphine in the brain demonstrates that the reduction of opioids in the brain may reduce the reward associated with these compounds.

### Opioid tolerance and dependence

One of the most challenging aspects of prolonged treatment with opioids is the progressive loss of efficacy referred to as opioid tolerance. Opioid tolerance is defined by Yaksh and Wallace as the reduction of analgesic efficacy of a particular dose of an opioid as that dose is repeatedly given over time [19]. Opioid tolerance occurs in as little as 2 weeks [67]. Tolerance is observable at the level of reduced analgesic and sedative effects. At the level of the cell, adenyl cyclase activity is disinhibited [68]. Research regarding the effect of chronic morphine exposure on the BBB is sparse. Whole brain and larger cortical blood vessels show an increase in expression of genes in the Mdr family including P-gp [69, 70]. These changes are correlated with decreased CNS uptake of morphine in rodents [69, 70]. Two studies suggest that the NMDA receptor signaling through the cyclooxygenase 2 pathway is involved in P-gp upregulation by morphine [69, 71], however, additional work is needed to understand the mechanistic details. Different physiological responses to opioids develop tolerance at different rates [19]. The constriction of the pupil (pupillary miosis) is an example of a response with little development of tolerance. Analgesia, sedation, respiratory depression, and constipation are examples of responses to which tolerance will build at a slower, more moderate pace. Cross-tolerance between different opioids can occur, but this is not always the case, suggesting small but meaningful differences in the action of different types of opioid agonists. Tolerance is reversible and suspension of administration of the drug will, over time, return efficacy of a particular dose to the original, basal levels.

Chronic administration of opioids will also lead to the development of a state of dependence. Dependence presents as a state in which cessation of opioid use, or administration of an opioid receptor antagonist such as naloxone or naltrexone, will result in the precipitation of withdrawal syndrome symptoms. Because opioids are an inhibitory signal to the cell, cells will increase signaling to compensate and return to normal function. Removing the inhibitory signal will result in an overactivation of affected cellular pathways leading to a variety of symptoms caused by the overactivation of the somatomotor cortex and autonomic nervous system [19]. Work by Nakagawa et al. showed that a glutamate transport activator, MS-153, was sufficient to prevent opioid dependence and withdrawal, suggesting glutamate may play a role in the formation of opioid dependence and withdrawal [72]. A study by Chaves et al. described that in the case of naloxone precipitated opioid withdrawals following sub-chronic morphine exposure, there was little change on P-gp at the BBB [73]. The major physical symptoms of withdrawal syndrome include diarrhea, vomiting, agitation, hyperalgesia, hyperthermia and hypertension. Feelings of depression, dysphoria and anxiety are also associated with withdrawal. Due to the fact these symptoms are highly aversive, prevention of withdrawal can act as a major motivator to continue use of the drug. This incentive to continue use can lead to overuse of, abuse of and addiction to opioids [74].

Tolerance to the euphoric effects of opioids develops rapidly and at a rate higher than many other effects [19]. Diminishing euphoria means users seeking this feeling are prone to ingesting a dose which can elicit a dangerous effect from a different response with a slower rate of tolerance. Because of this and severe withdrawal symptoms, addiction and abuse are problems for many individuals including both those who began as therapeutic users and exclusively recreational users [75]. Opioid addiction, also known as opioid use disorder, is a psychological condition defined as “compulsive, prolonged self-administration of opioid substances that are used for no legitimate medical purpose or, if another medical condition is present that requires opioid treatment, that are used in doses greatly in excess of the amount needed for that medical condition,” [76]. Both those using opioids recreationally for euphoric effects and those who begin using them for medical conditions are at risk of addiction. Tolerance, dependence and the risk of addiction should be considered when prescribing opioids for post-surgical pain management.

The presence of a mental health condition can increase the likelihood of substance abuse. As many as 50% of patients with dipolar disorder have been found to have a substance abuse problem at some time in their life [77]. A survey by Martins et al. showed that several psychopathologies, especially anxiety disorders and bipolar I disorder, are associated with an increased incidence of opioid use [78]. This was an increased risk for those with a pre-existing condition as well as in individuals with newly diagnosed disorders in which the patient had a history of non-medical opioid use. This study suggests individuals with anxiety disorders and with bipolar I disorder will use opioids as a means of “self-medication”. In a disease like bipolar disorder with many different presenting episodes, the use of heroin is consistent across all types of episodes [79]. The use of opioids as a “self-medication” is a major public health concern. The comorbidity of substance abuse disorders and other psychopathologies demonstrates that this population must be treated with increased care and attention.

### An epidemic

Opioid abuse has reached epidemic proportions in the United States. This has raised the awareness of opioid abuse as a public health issue. Several states have increased funding for treatment of opioid dependence to combat the trends of increased abuse and overdose deaths [1, 80]. However, there is insufficient treatment capacity to address the opioid dependence problem [1]. Cost of treatment is a challenge that significantly impacts the ability to increase capacity; approximately 25 billion dollars was spent in 2007 on extra healthcare costs related to opioid abuse [81]. New affordable, effective treatments and government funding for these programs will be essential to changing these trends.

Multiple societal, physiological and psychological factors contribute to the increasing opioid abuse. A majority of modern recreational opioid users begin their experience with opioids as therapeutics [82]. A study of patients diagnosed with opioid abuse disorder showed that almost 80% of these patients had a prescription for opioids before the first diagnosis of opioid abuse [83]. This study was also able to show that of the 20% that did not have a previous prescription, over half of them had a close family member who had a prescription before the first diagnosis of opioid abuse. This suggests that the availability of opioids from a family member can be a risk factor for abuse. Misuse of prescription refills and “doctor shopping,” a situation where an individual seeking opioids may go to several different doctors to receive multiple prescriptions for the drugs, are common problems associated with prescribed opioids [12, 84, 85]. Use of online pharmacies, some of which require little documentation, and the dark web system of encrypted websites which is designed to allow the user complete anonymity has opened the door for illicit sale of prescription opioids [86]. Early refills are a subset of prescription abuse that requires additional scrutiny [87]. Some chronic opioid users increase use because opioids lose analgesic effectiveness over time, and the patient may resort to taking more pills to manage pain [88]. However, in other cases the additional pills are given to others or sold.

Physicians prescribing opioids must be examined as a factor contributing to the opioid epidemic, but must also be part of the solution of the problem. Physicians prescribe opioids at different rates due to many factors including: patient satisfaction surveys online [89]; professional repercussions for using (or not using) prudent judgment [89]; and how concerned a physician is about opioids as a public health problem (physicians less concerned with opioids as a problem are more likely to have patients on long-term opioid therapy for chronic pain as well [90]). Since 2014, the changing opinions of physicians towards opioids caused a decrease in the number of opioid prescriptions dispensed in the United States relative to predicted rates [91]. While these rates have dropped, the overall opioid epidemic has not changed [92].

Several studies suggest that a switch from prescription opioids to heroin is fueling the opioid epidemic. Heroin use has increased in the United States over the last decade [93]. This is likely due to the increase in popularity of opioid pain pills. A review of surveys interviewing heroin users who used opioid pain pills before the first time the individual used heroin range from 40 to 86% but was enough to suggest a relationship [82]. From 2010 to 2013, individuals who had used an opioid in the past month began to use only prescription opioids less and used a combination of opioids and heroin more, according to a self-administered survey of diagnosed opioid abusers [94]. The availability of heroin in the United States is increasing [95]. This report also states heroin is less expensive than prescription opioids on the streets. The estimated cost of a 10 mg dose of oxycodone is approximately $10 while it is estimated 50 mg of 50% pure heroin is around the same price. Heroin use may also be favorable because of the increased potency of the drug compared to morphine; a larger amount of heroin is able to cross the BBB compared to morphine [27]. Addressing this epidemic requires: (1) the development of better options for treating pain that takes into account the necessity of crossing the BBB to elicit an effect; (2) the societal and political will to develop strategies to combat the problem; (3) increased capacity to treat opioid dependence; and (4) a change in attitude such that opioid addiction is viewed as a medical problem rather than a criminal offense.

## Conclusion

Opioids are a powerful tool for the treatment of pain. Effective and responsible clinical use of opioids and their derivatives is complicated by P-gp at the BBB, tolerance and dependence. For the treatment of short/moderate duration post-surgical pain the analgesic benefit must be balanced with the risk of dependence, addiction and abuse. Regulation of opioid access to the CNS by the blood brain barrier is central to the ability of currently available opioids to alleviate pain, but also to induce euphoria. This BBB effect contributes to the addiction and abuse that is fueling the opioid epidemic.

Continued research to develop new strategies and agents to alleviate pain is required. Some strategies, such as the development of opioid derivatives that act locally show promise in pre-clinical models [96]. The basis of this strategy is using the inherent challenges associated with designing therapeutics that will cross the blood–brain barrier to design opioid-based treatment strategies so that the opioids do not cross the BBB. Peripherally acting opioid analgesics are generally free from the addictive nature of traditional centrally acting opioid analgesics [97]. This type of analgesic was traditionally thought to be less effective, but there is increasing evidence this may be a promising strategy for pain management under certain conditions [98]. A recent study by Spahn et al. demonstrated that computer modeling could be used to design a novel therapeutic effective at relieving pain without exhibiting addiction potential [96]. The opioid fentanyl was fluorinated resulting in selection for mu opioid receptors in environments with lower pH, such as those associated with inflamed tissue. The modified fentanyl demonstrated no addictive properties in a conditioned place preference test. Because of the power of computer based research in receptor affinities and the increasingly complex computer modeling systems, this approach may represent a way to modify already available opioid analgesics. Alternative routes of administration of already existing opioids are also showing promise. A study by Arti and Mehdinsab demonstrated that an intra-articular injection of opioid analgesics reduced pain following arthroscopic surgery compared to control [99]. This study demonstrated this effect using a variety of different opioid analgesics including: morphine, methadone, pethidine, and tramadol. By demonstrating analgesia can be achieved by multiple opioid analgesics in this way, this study demonstrated the potential the peripheral opioid system has in analgesia. An advantage to this approach is it can be performed with already available opioid analgesics. This route of administration is selective in nature and works only in inflamed tissue, similar to the previously described study [100]. An understanding of the BBB and how it can be used to keep opioids out of the CNS combined with further study into peripheral action of opioid analgesics, represents a potential new path into systemically administered opioids that only act in inflamed or painful areas without the unwanted side effects of dependence or addiction.

The opioid epidemic has sparked renewed interest in non-opioid-based pain treatment strategies. An extensive discussion of these approaches to pain treatment/management are beyond the scope of this review. However, some strategies with clinical promise include: identification of alternate pain pathways that can be targeted by therapeutics [101, 102]; use of non-opioid drugs [103, 104]; first line treatment of pain with physical therapy [105]; and development innovative alternatives such as the use of green light [106]. Dealing with the opioid epidemic, however, is more complex than just developing novel pain treatments. It will also require: responsible use of opioids where medically warranted; acceptance of these new treatment options by patients and insurance companies; and funding for opioid addiction treatment combined with social and political changes.

## Abbreviations

BBB: blood–brain barrier
P-gp: P-glycoprotein
OIH: opioid induced hyperalgesia
PAG: periaqueductal grey
M3G: morphine-3-glucuronide
M6G: morphine-6-glucuronide
6-MAM: 6-monoacytylmorphine
EDDP: 2-ethylidene-1,5-dimethyl-3,3-diphenylpyrrolidine
CNS: central nervous system

## References

[1] United States Department of Health and Human Services. The opioid epidemic: by the numbers. 2016;60. http://www.hhs.gov/sites/default/files/Factsheet-opioids-061516.pdf. Accessed 6 June 2016.

[2] Rudd RA, Aleshire N, Zibbell JE, Gladden RM (2016). Increases in drug and opioid overdose deaths—United States, 2000–2014. MMWR Morb Mortal Wkly Rep.

[3] Joshi GP, Beck D, Emerson R, Halaszynki T, Jahr J, Lipman A (2014). Defining new directions for more effective management of surgical pain in the United States: highlights of the inaugural surgical pain congress ä. Highlights Inaug Surg Pain Congr.

[4] Basbaum AI, Bautista DM, Scherrer G, Julius D (2009). Cellular and molecular mechanisms of pain. Cell.

[5] Woolf CJ, Ma Q (2007). Nociceptors–noxious stimulus detectors. Neuron.

[6] Brownstein MJ (1993). A brief history of opiates, opioid peptides, and opioid receptors. Proc Natl Acad Sci USA.

[7] Schiff PL (2002). Opium and its alkaloids. Am J Pharm Educ.

[8] Sertuner F. Trommsdorff’s J Pharm. 1806;47–93.

[9] Wright CRA. On the action of organic acids and their anhydrides on the natural alkaloids. Part I. J Chem Soc. 1872;27:1031–43.

[10] Bockmuhl VM, Ehrhart G. Uber eine neue Klasse von spasmolytisch und aiialgetisch wirkenden Verbindungen. I. Eur J Org Chem. 1947;561:52–85.

[11] Fugelstad A, Stenbacka M, Leifman A, Nylander M, Thiblin I (2007). Methadone maintenance treatment: the balance between life-saving treatment and fatal poisonings. Addiction.

[12] Wilkerson RG, Kim HK, Windsor TA, Mareiniss DP (2016). The opioid epidemic in the United States. Emerg Med Clin N Am.

[13] Porter J, Jick H (1980). Addiction rare in patients treated with narcotics. N Engl J Med.

[14] Zenz M, Strumpf M, Tryba M (1992). Long-term oral opioid therapy in patients with chronic nonmalignant pain. J Pain Symptom Manage.

[15] Portenoy RK, Foley KM (1987). Chronic use of opioid analgesics in non-malignant pain [letter]. Pain.

[16] Weingarten MA (1991). Chrnoic opioid therapy in patients with a remote history of substance abuse. J Pain Symptom Manage.

[17] Okie S (2010). A Flood of Opioids, a Rising Tide of Deaths. New England journal. N Engl J Med.

[18] Cantrill SV, Brown MD, Carlisle RJ, Delaney KA, Hays DP, Nelson LS (2012). Clinical policy: critical issues in the prescribing of opioids for adult patients in the emergency department. Ann Emerg Med.

[19] Yaksh TL, Wallace MS. Chapter 18 : opioids, analgesia, and pain management. In: Goodman and Gilman’s: the pharmacological basis of therapeutics, 12th ed. New York: McGraw-Hill; 2011.

[20] Osbourne R, Joel S, Trew D, Slevin M (1988). analgesic activity of morphine-6-glucuronide. Lancet.

[21] Smith MT (2000). Neuroexcitatory effects of morphine and hydromorphone: evidence implicating the 3-glucuronide metabolites. Clin Exp Pharmacol Physiol.

[22] Sia AT, Lim Y, Lim ECP, Goh RWC, Law HY, Landau R (2008). A118G single nucleotide polymorphism of human mu-opioid receptor gene influences pain perception and patient-controlled intravenous morphine consumption after intrathecal morphine for postcesarean analgesia. Anesthesiology.

[23] Yaksh TL (1997). Pharmacology and mechanisms of opioid analgesic activity. Acta Anaesthesiol Scand.

[24] Bundgaard M, Abbott NJ (2008). All vertebrates started out with a glial blood–brain barrier 4–500 million years ago. Glia.

[25] Mayer F, Mayer N, Chinn L, Pinsonneault RL, Bainton RJ (2011). Evolutionary conservation of vertebrate blood–brain barrier chemoprotective mechanisms in *Drosophila*. J Neurosci.

[26] Mahringer A, Ott M, Fricker G. The blood brain barrier (BBB). Heidelberg: Springer; 2014. p. 1–20.

[27] Seleman M, Chapy H, Cisternino S, Courtin C, Smirnova M, Schlatter J (2014). Impact of P-glycoprotein at the blood–brain barrier on the uptake of heroin and its main metabolites: behavioral effects and consequences on the transcriptional responses and reinforcing properties. Psychopharmacology.

[28] Campbell AW (2016). The blood–brain barrier. Altern Ther.

[29] Nakagawa S, Deli MA, Kawaguchi H, Shimizudani T, Shimono T, Kittel Á (2009). A new blood–brain barrier model using primary rat brain endothelial cells, pericytes and astrocytes. Neurochem Int.

[30] Abbott NJ, Patabendige AAK, Dolman DEM, Yusof SR, Begley DJ (2010). Structure and function of the blood–brain barrier. Neurobiol Dis.

[31] Dick AP, Harik SI, Klip A, Walker DM (1984). Identification and characterization of the glucose transporter of the blood–brain barrier by cytochalasin B binding and immunological reactivity. Proc Natl Acad Sci USA.

[32] Jones PM, George AM (2004). The ABC transporter structure and mechanism: perspectives on recent research. Cell Mol Life Sci.

[33] Cordon-Cardo C, O’Brien JP, Casals D, Rittman-Grauer L, Biedler JL, Melamed MR (1989). Multidrug-resistance gene (P-glycoprotein) is expressed by endothelial cells at blood–brain barrier sites. Proc Natl Acad Sci USA.

[34] Schinkel AH, Smit JJM, van Tellingen O, Beijnen JH, Wagenaar E, van Deemter L (1994). Disruption of the mouse mdr1a P-glycoprotein gene leads to a deficiency in the blood–brain barrier and to increased sensitivity to drugs. Cell.

[35] Bostrom E, Hammarlund-Udenaes M, Simonsson US (2008). Blood–brain barrier transport helps to explain discrepancies in in vivo potency between oxycodone and morphine. Anesthesiology.

[36] Ambudkar SV, Kim I, Sauna ZE (2006). The power of the pump: mechanisms of action of P-glycoprotein (ABCB1). Eur J Pharm Sci.

[37] Thomas H, Coley H (2003). Overcoming multidrug resistance in cancer: an update on the clinical strategy of inhibiting p-glycoprotein. Cancer Control.

[38] Liang X, Aszalos A (2006). Multidrug transporters as drug targets. Curr Drug Targets.

[39] Yamada H, Ishii K, Ishii Y, Ieiri I, Nishio S, Morioka T (2003). Formation of highly analgesic morphine-6-glucuronide following physiologic concentration of morphine in human brain. J Toxicol Sci.

[40] Tournier N, Chevillard L, Megarbane B, Scherrmann J, Pirnay S, Decleves X (2010). Interaction of drugs of abuse and maintenance treatments with human P-glycoprotein (ABCB1) and breast cancer resistance protein (ABCG2). Int J Neurophychopharmacology..

[41] Fujita KI, Ando Y, Yamamoto W, Miya T, Endo H, Sunakawa Y (2010). Association of UGT2B7 and ABCB1 genotypes with morphine-induced adverse drug reactions in Japanese patients with cancer. Cancer Chemother Pharmacol.

[42] De Gregori S, De Gregori M, Ranzani GN, Allegri M, Minella C, Regazzi M (2012). Morphine metabolism, transport and brain disposition. Metab Brain Dis.

[43] Bourasset F, Cisternino S, Temsamani J, Scherrmann J (2003). Evidence for an active transport of morphine-6-β-d-glucuronide but not P-glycoprotein-mediated at the blood–brain barrier. J Neurochem.

[44] Balayssac D, Cayre A, Ling B, Maublant J, Penault-Llorca F, Eschalier A (2009). Increase in morphine antinociceptive activity by a P-glycoprotein inhibitor in cisplatin-induced neuropathy. Neurosci Lett.

[45] Zelcer N, van de Wetering K, Hillebrand M, Sarton E, Kuil A, Wielinga PR (2005). Mice lacking multidrug resistance protein 3 show altered morphine pharmacokinetics and morphine-6-glucuronide antinociception. Proc Natl Acad Sci USA.

[46] Wandel C, Kim R, Wood M, Ch MBB, Wood A, Ch MBB (2002). Interaction of morphine, fentanyl, sufentanil, alfentanil, and loperamide with the efflux drug transporter P-glycoprotein. Anesthesiology.

[47] Montesinos RN, Moulari B, Gromand J, Beduneau A, Lamprecht A (2014). Coadministration of P-glycoprotein modulators on loperamide pharmacokinetics and brain distribution. Drug Metab Dispos.

[48] Regnard C, Twycross R, Mihalyo M, Wilcock A (2011). Loperamide. J Pain Symptom Manag.

[49] Schinkel AH, Wagenaar E, Mol CAAM, Van Deemter L (1996). P-Glycoprotein in the blood–brain barrier of mice influences the brain penetration and pharmacological activity of many drugs. J Clin Invest.

[50] Kaiko RF, Wallenstein SL, Rogers A (1981). Relative analgesic potency of intramuscular heroin and morphine in cancer patients with postoperative pain and chronic pain due to cancer. NIDA Res Minigr Ser.

[51] Selley DE, Cao CC, Sexton T, Schwegel JA, Martin TJ, Childers SR (2001). μ opioid receptor-mediated G-protein activation by heroin metabolites: evidence for greater efficacy of 6-monoacetylmorphine compared with morphine. Biochem Pharmacol.

[52] Boix F, Andersen JM, Mørland J (2013). Pharmacokinetic modeling of subcutaneous heroin and its metabolites in blood and brain of mice. Addict Biol..

[53] Boström E, Simonsson USH, Hammarlund-Udenaes M (2005). Oxycodone pharmacokinetics and pharmacodynamics in the rat in the presence of the P-glycoprotein inhibitor PSC833. J Pharm Sci.

[54] Silvasti M, Rosenburg P, Seppala T, Svartling N, Pitkanen M (1998). Comparison of analgesic efficacy of oxycodone and morphine in postoperative intravenous patient-controlled analgesia. Acta Anaesthesiol Scand.

[55] Bostrom E, Simonsson USH, Hamarlund-Udenases M (2006). In vivo blood–brain barrier transport of oxycodone in the rat: indications for active influx and implications for pharmacokinetics/pharmacodynamics. Drug Metab Dispos.

[56] Okura T, Hattori A, Takano Y, Sato T, Hammarlund-udenaes M, Terasaki T (2008). Involvement of the pyrilamine transporter, a putative organic cation transporter, in blood–brain barrier transport of oxycodone. Drug Metab Dispos..

[57] Hagen NA, Wasylenko E (1999). Methadone: outpatient titration and monitoring strategies in cancer patients. J Pain Symptom Manage.

[58] Wang JS, Ruan Y, Taylor RM, Donovan JL, Markowitz JS, DeVane CL (2004). Brain penetration of methadone (*R*)- and (*S*)-enantiomers is greatly increased by P-glycoprotein deficiency in the blood–brain barrier of Abcb1a gene knockout mice. Psychopharmacology.

[59] Sullivan HR, Due SL (1973). Urinary metabolites of *dl*-methadone in maintenance subjects. J Med Chem.

[60] Mccance-katz EF (2011). (*R*)-methadone versus racemic methadone: what is best for patient care?. Addiction.

[61] Crettol S, Digon P, Powell Golay K, Brawand M, Eap CB (2007). In vitro P-glycoprotein-mediated transport of (*R*)-, (*S*)-, (*R*,*S*)-methadone, LAAM and their main metabolites. Pharmacology.

[62] Mercer SL, Coop A (2011). Opioid analgesics and P-glycoprotein efflux transporters: a potential systems-level contribution to analgesic tolerance. Curr Top Med Chem.

[63] Volpe DA, Mcmahon GA, Mellon RD, Katki AG, Parker RJ, Colatsky T (2011). Uniform assessment and ranking of opioid Mu receptor binding constants for selected opioid drugs. Regul Toxicol Pharmacol.

[64] Henthorn TK, Liu Y, Mahapatro M, Ng K (1999). Active transport of fentanyl by the blood–brain barrier 1. J Pharmacol Exp Ther.

[65] Klepstad P, Dale O, Skorpen F, Borchgrevink PC, Kaasa S (2005). Genetic variability and clinical efficacy of morphine. Acta Anaesthesiol Scand.

[66] Xi Z, Stein EA (2002). GABAergic mechanisms of opiate reinforcement. Alcohol Alcohol.

[67] Doi K, Gibbons G (2014). Improving postoperative pain management in orthopedic total joint surgical patients with opioid tolerance using the iowa model of evidence-based practice. ASPAN Natl Conf Abstr.

[68] Pasternak GW (2005). Molecular biology of opioid analgesia. J Pain Symptom Manage.

[69] Yousif S, Saubamea B, Cisternino S, Marie-Claire C, Dauchy S, Scherrmann J-M (2008). Effect of chronic exposure to morphine on the rat blood–brain barrier: focus on the P-glycoprotein. J Neurochem.

[70] Aquilante CL, Letrent SP, Pollack GM, Brouwer KL (2000). Increased brain P-glycoprotein in morphine tolerant rats. Life Sci.

[71] Li Y, Yue H, Xing Y, Sun H, Pan Z, Xie G (2010). Oxymatrine inhibits development of morphine-induced tolerance associated with decreased expression of P-glycoprotein in rats. Integr Cancer Ther.

[72] Nakagawa T, Ozawa T, Shige K, Yamamoto R, Minami M (2001). Inhibition of morphine tolerance and dependence by MS-153, a glutamate transporter activator. Eur J Pharmacol.

[73] Chaves C, Gomez-Zepeda D, Auvity S, Menet M-C, Crete D, Labat L (2015). Effect of subchronic intravenous morphine infusion and naloxone-precipitated morphine withdrawal on P-gp and Bcrp at the rat blood–brain barrier. J Pharm Sci.

[74] Kahan M, Srivastava A, Wilson L, Gourlay D, Midmer D (2006). Misuse of and dependence on opioids: study of chronic pain patients. Can Fam Physician..

[75] Gupta A, Christo PJ (2009). Opioid abuse and dependence. J Nurse Pract.

[76] Author. Substance-related and addictive disorders. In: Diagnostic and statistical manual of mental disorders. 5th ed. Washington, DC: American Psychiatric Association; 2013.

[77] Sonne S, Brady K (1999). Substance abuse and bipolar comorbidity. Psychiatr Clin N Am.

[78] Martins SS, Keyes KM, Storr CL, Zhu H, Chilcoat HD (2009). Pathways between nonmedical opioid use/dependence and psychiatric disorders: results from the National Epidemiologic Survey on Alcohol and Related Conditions. Drug Alcohol Depend.

[79] Maremmani I, Giovanni A, Maremmani I, Rugani F, Rovai L, Pacini M (2012). Clinical presentations of substance abuse in bipolar heroin addicts at time of treatment entry. Ann Gen Psychiatry.

[80] Executive Office of Health and Humand Serivices. Recovery Support. http://www.mass.gov. 2017. http://www.mass.gov/eohhs/gov/departments/dph/programs/substance-abuse/recovery-support.html. Accessed 1 Jan 2017.

[81] Birnbaum HG, White AG, Schiller M, Waldman T, Cleveland JJM, Setnik B (2010). Societal costs of opioid abuse, dependence and misuse in the United States. Value Health.

[82] Kanouse AB, Compton P (2015). The epidemic of prescription opioid abuse, the subsequent rising prevalence of heroin use, and the federal response. J Pain Palliat Care Pharmacother.

[83] Shei A, Rice JB, Kirson NY, Bodnar K, Birnbaum HG, Holly P (2015). Sources of prescription opioids among diagnosed opioid abusers. Curr Med Res Opin.

[84] McDonald DC, Carlson KE (2013). Estimating the prevalence of opioid diversion by “doctor shoppers” in the United States. PLoS ONE.

[85] Han H, Kass PH, Wilsey BL, Li C-S (2014). Increasing trends in Schedule II opioid use and doctor shopping during 1999–2007 in California. Pharmacoepidemiol Drug Saf.

[86] Aldridge J, Decary-Hetu D (2016). Hidden wholesale: the drug diffusing capacity of online drug cryptomarkets. Int J Drug Policy.

[87] Lange A, Lasser KE, Xuan Z, Khalid L, Beers D, Heymann OD (2015). Variability in opioid prescription monitoring and evidence of aberrant medication taking behaviors in urban safety-net clinics. Pain.

[88] Katz N, Panas L, Kim M, Audet A, Bilansky A, Eadie J (2010). Usefulness of prescription monitoring programs for surveillance—analysis of Schedule II opioid prescription data in Massachusetts, 1996–2006. Pharmacoepidemiol Drug Saf.

[89] Lembke A (2012). Why doctors prescribe opioids to known opioid abusers. N Engl J Med.

[90] Wilson HD, Dansie EJ, Kim MS, Moskovitz BL, Chow W, Turk DC (2013). Clinicians’ attitudes and beliefs about opioids survey (CAOS): instrument development and results of a national physician survey. J Pain.

[91] Jones CM, Lurie PG, Throckmorton DC (2017). Effect of US drug enforcement administration’s rescheduling of hydrocodone combination analgesic products on opioid analgesic prescribing. J Am Med Assoc Intern Med.

[92] Kertesz S (2017). Turning the tide or riptide? The changing opioid epidemic. Subst Abus.

[93] Center for Behavioral Health Statistics and Quality. Behavioral health trends in the United States: results from the 2014 national survey on drug use and health. 2015. http://www.samhsa.gov/data/sites/default/files/NSDUH-FRR1-2014/NSDUH-FRR1-2014.pdf%5Cn, http://www.samhsa.gov/data/. Accessed 23 Dec 2016.

[94] Cicero TJ, Ellis MS, Harney J (2015). Shifting patterns of prescription opioid and heroin abuse in the United States. N Engl J Med.

[95] The Office of the President of the United Sates. National drug control strategy. 2015. https://www.whitehouse.gov//sites/default/files/ondcp/policy-and-research/2015_national_drug_control_strategy_0.pdf. Accessed 23 Dec 2016.

[96] Spahn V, Massaly N, Temp J, Durmaz V, Sabri P, Reidelbach M (2017). A nontoxic pain killer designed by modeling of pathological receptor conformations. Science (80 −).

[97] Kieffer BL, Gavériaux-ruff C (2002). Exploring the opioid system by gene knockout. Prog Neurobiol.

[98] Oeltjenbruns J, Schäfer M (2005). Peripheral opioid analgesia: clinical applications. Anesth Tech Pain Manag..

[99] Arti H, Mehdinasab SA (2011). The comparison effects of intra-articular injection of different opioids on postoperative pain relieve after arthroscopic anterior cruciate ligament reconstruction: a randomized clinical trial study. J Res Med Sci..

[100] Reuben SS, Sklar J (2000). Current concepts review pain management in patients who undergo outpatient arthroscopic surgery of the knee. J Bone Jt Surg.

[101] Ding H, Czoty PW, Kiguchi N, Cami-kobeci G, Sukhtankar DD, Nader MA (2016). A novel orvinol analog, BU08028, as a safe opioid analgesic without abuse liability in primates. Proc Natl Acad Sci USA..

[102] Emery EC, Luiz AP, Wood JN (2016). Nav1.7 and other voltage-gated sodium channels as drug targets for pain relief. Expert Opin Ther Targets..

[103] Hill KP, Palastro MD, Johnson B, Ditre JW (2017). Cannabis and pain: a clinical review. Cannabis Cannabinoid Res..

[104] Ong CKS, Seymour RA, Lirk P, Merry AF (2010). Combining paracetamol (acetaminophen) with nonsteroidal antiinflammatory drugs: a qualitative systematic review of analgesic efficacy for acute postoperative pain. Anesth Analg.

[105] Hayden JA, Van Tulder MW, Tomlinson G (2005). Systematic review: strategies for using exercise therapy to improve outcomes in chronic low back pain. Ann Intern Med.

[106] Ibrahim MM, Patwardhan A, Gilbraith KB, Moutal A, Yang X, Chew LA (2017). Long-lasting antinociceptive effects of green light in acute and chronic pain in rats. Pain..

